# Aging Resources and Vulnerability Across Targets: Self-Other Profiles in Israeli and Japanese Assisted-Living Residents

**DOI:** 10.1007/s10823-026-09595-8

**Published:** 2026-09-08

**Authors:** Ehud Bodner, Sara Cohen-Fridel, Klaus Rothermund, Maria Clara de Paula Couto, Yasuko Ishimoto, Ryota Sakamoto, Michiko Fujisawa, Kiichi Hirayama, Tamar Gitlitz, Arie Rotshtein, Hadas Kushelevich, Taizo Wada

**Affiliations:** 1 Department of Social and Health Sciences and Music Department, Bar-Ilan University, Ramat Gan, Israel; 2 Department of Social and Health Sciences, Bar-Ilan University, Ramat Gan, Israel; 3 Institute of Psychology, Friedrich Schiller University Jena, Jena, Germany; 4 Kawasaki University of Medical Welfare, Kurashiki, Japan; 5 Center for Southeast Asian Studies, Kyoto University, Kyoto, Japan; 6 Faculty of Medicine, Kyoto University, Kyoto, Japan; 7 School of Social Work, Tel Aviv University, Tel Aviv, Israel; 8 Institute for Liberal Arts, Doshisha University, Kyoto, Japan

**Keywords:** Age stereotypes, Cultural differences, Independence, Interdependence, Views on aging

## Abstract

Views on aging (VoA) are multidimensional: they may differ by target (the aging self vs. older adults in general) and by evaluative content (resources vs. vulnerability). However, little is known about how these facets are configured across cultural contexts, or whether other-directed VoA are linked differently to personal self-views in different countries. In a cross-sectional two-country comparison, we analyzed 390 assisted-living records from Japan (*n* = 203) and Israel (*n* = 187), using brief, parallel VoA measures together with measures of independence, interdependence, and background characteristics. Japanese participants reported higher interdependence than Israeli participants, whereas independence, which was examined non-directionally, did not differ significantly. Country differences in VoA were profile-specific rather than uniform: Israeli participants scored higher on Self-Resources, Other-Resources, and Other-Vulnerability, whereas Self-Vulnerability did not differ between countries. The raw Other-Resources–Self-Resources correlation was significantly stronger in Japan than in Israel, and the focused adjusted moderation model likewise indicated a steeper association in Japan. Because the design was cross-sectional and several brief scales showed modest reliability, the findings can only indicate country-level patterning and associations rather than demonstrate a cultural mechanism. The results support treating VoA as a target- and content-specific profile rather than as a single evaluative score.

## Introduction

### Views of Aging as Multidimensional Profiles

Contemporary work on views of aging (VoA) emphasizes that this umbrella term is not reducible to a single evaluative dimension but comprises multiple components that may operate differently across individuals and cultural contexts (Kornadt et al., 2020; Shrira et al., 2022). Conceptual accounts distinguish VoA directed toward the self as an aging person from views directed toward older adults in general and further recognize that each target may include positive and negative content (Rothermund & de Paula Couto, 2024; Shrira et al., 2022). In the present study, resource-focused VoA refers to attributions of capabilities and assets in later life, whereas vulnerability-focused VoA refers to attributions of exposure to loss, dependence, or social disconnection. These contents need not be opposite endpoints of one continuum: individuals and cultural contexts may acknowledge both resources and vulnerabilities. Crossing evaluative content (resources vs. vulnerability) with target (self vs. other) therefore yields a four-facet profile. A global positivity score could conceal offsetting patterns, whereas the four-facet framework can identify whether cross-national differences are concentrated in a particular target or content and whether other-directed representations are linked to personal aging self-views. The distinction is compatible with contemporary multidimensional VoA frameworks and with adjacent lifespan perspectives on gains, losses, strengths, vulnerabilities, and resources (Charles, 2010; Hobfoll et al., 2018), although the present brief scales are descriptive VoA measures rather than direct operationalizations of strength and vulnerability integration or conservation-of-resources theory (Diehl & Wahl, 2024; Kornadt et al., 2020).

VoA are relevant beyond attitudinal description because general age stereotypes and personal aging self-perceptions have been associated prospectively with physical and mental health, and gain- and loss-related self-perceptions may have different implications for these outcomes (Brothers et al., 2021). Assisted-living settings provide a particularly informative context because autonomy, participation, capability, security, social connectedness, and dependence are salient features of residents’ everyday experience (Jonasson et al., 2023).

Studying residents in broadly comparable assisted-living settings in Israel and Japan therefore permits the examination of resource- and vulnerability-related VoA and of self- and other-directed aging views in a context where independence, support needs, and the negotiation of autonomy are particularly salient, and where socially shared views of aging may become personally self-relevant (Ball et al., 2004; Diehl & Wahl, 2024; Levy, 2009). At the same time, the middle- to high-socioeconomic-status facilities sampled here restrict the generalizability of the findings.

### Cultural Self-Construal Framework

In cultural psychology, a fundamental and well-documented distinction is drawn between independent and interdependent (context-dependent/relational) self-construal. The core assumption is that cultures systematically emphasize different aspects of the self and social relationships (Kitayama & Salvador, 2024; Markus & Kitayama, 1991; Oyserman et al., 2002; Singelis, 1994; Vignoles et al., 2016). One consequence of differences in these aspects of the self and their relation to society is that cross-cultural variation can be expected in the extent to which individuals experience themselves as embedded in social ties and oriented toward norms of adjustment, fitting in, and belonging. This perspective on the independent and interdependent self-construal (Markus & Kitayama, 1991) has served as the basis for extensive empirical measurement of these two self-construal dimensions, which differentiate independence and interdependence as distinct societal dimensions.

The distinction between the independent and interdependent self-construals has been useful for cultural psychology because it provides an applicable framework for theorizing how cultures shape the individual’s self-definitions and social behavior (Kitayama & Salvador, 2024; Markus & Kitayama, 1991). In the present comparison, Israel can be situated cautiously as a relatively Western-modern context in which self-reported individualism has been documented in Israeli society (notably among secular groups; Sagy et al., 1999; Sagy et al., 2001), while Japan represents an East Asian cultural context in which social connectedness and orientation toward group norms have been theorized as central features of selfhood (Markus & Kitayama, 1991). Convergent evidence for a possible difference between Israel and Japan comes from a U.S.–Japan comparison showing that interdependence was significantly higher among older Japanese than older Americans, underscoring the plausibility of expecting higher interdependence in Japan in aging-related samples (Levy et al., 2009). Although the comparison is not Israel–Japan, it aligns with the broader self-construal framework in which Japan is characterized by stronger norm-oriented relationality than Western contexts (Kitayama & Salvador, 2024; Markus & Kitayama, 1991; Singelis, 1994; Vignoles et al., 2016). On this basis, it is expected that Japanese older adults will report higher levels of interdependence than Israeli older adults. Because cross-cultural self-report means can be affected by response styles and reference-group processes, we treat self-construal as a contextual anchor and focus our primary tests on VoA profiles and self–other coupling rather than on fine-grained mean-level inferences from self-construal scores.

With this anchoring in mind, by contrast, a symmetrical opposed hypothesis about independence is less straightforward. The Japanese context is characterized by documented historical increases in individualism-related indicators alongside persisting collectivistic elements, suggesting that independence may be more cohort- and modernization-contingent, particularly for cohorts born during or shortly after World War II and socialized through postwar reconstruction and rapid modernization (Ogihara, 2017, 2018). Consistent with this complexity, and with the failure to support the contention that Japan is more collectivistic and less individualistic than the United States (Takano & Osaka, 2018), we regard independence as an open, non-directional empirical question in the present study, while hypothesizing more interdependence in older Japanese than in older Israelis.

Building on this expectation of stronger interdependence in Japan than in Israel, we now consider how country differences in VoA may be configured. More specifically, we explain why country differences are expected to emerge as a profile across the target (self vs. other) and valence (resources vs. vulnerability) dimensions, rather than as a single, uniform mean difference.

### Cross-National VoA Configuration

When examining comparisons of self-versus-other views of aging between Israel and Japan in the literature, one cannot find a specific, direct comparison. Nevertheless, several studies support the expectation that cross-national differences in views on aging can be treated as a profile, that is, a configuration that can differ across targets and valence, rather than as a single, uniform shift in overall positivity. First, cross-cultural evidence on 26 countries, including Israel and Japan, shows that VoA vary systematically across countries and are associated with culture-level characteristics, underscoring that national context can meaningfully pattern aging beliefs (Löckenhoff et al., 2009). Second, contemporary frameworks of VoA emphasize that they are multidimensional and can be directed toward older adults in general rather than toward the self as an older person, with these components not necessarily aligning (Shrira et al., 2022). These frameworks and the studies that follow them justify modeling self-perceptions and other-directed views as distinct targets rather than interchangeable indicators (Diehl & Wahl, 2024; Kornadt et al., 2020). Third, cross-cultural syntheses of attitudes toward older adults indicate that societal evaluations of older adults exhibit substantial heterogeneity across nations and operationalizations, making it less likely that any one culture is “more positive” or “more negative” across all aspects of aging (North & Fiske, 2015). Consistent with this point, evidence suggests that cross-cultural conclusions about whether East Asian contexts view older adults more positively depend strongly on the measures used, supporting the expectation that cultural differences may emerge in some facets of VoA but not in others (Vauclair et al., 2017; Voss et al., 2018). Taken together, these findings do not establish a specific Israel–Japan pattern for the four suggested facets of VoA (i.e., self/other and resources/vulnerability). However, they provide a sufficient basis to expect that country differences will be expressed differently when country (Japan vs. Israel), target (self vs. other), and valence (resources vs. vulnerability) are measured, rather than as a single main effect on an overall VoA score. Therefore, another hypothesis was that country differences in views on aging would be profile-specific rather than uniform, emerging as a significant Country × Target × Valence pattern rather than as a single overall mean difference.

### Age Norms Internalization

Following this hypothesis, we next examine whether the two countries also differ in the linkage between other-directed representations of aging and individuals’ self-perceptions of their own aging. A key theoretical reason to expect that the VoA of “older adults in general” and the VoA of “the self as old” are not merely similar judgments but may be dynamically connected through processes of internalization and self-relevance lies in Levy’s stereotype embodiment theory (Levy, 2009; see also Rothermund & Brandtstädter, 2003). Levy proposes that age stereotypes are absorbed from the surrounding culture across the life course and can become increasingly self-relevant as individuals age, thereby shaping how they interpret their own aging and potentially influencing their functioning and health (Levy, 2009). Although direct evidence on country differences in other–self coupling is scarce, Levy and colleagues (2009) conducted a U.S.–Japan comparison consistent with the reasoning developed here. They found that older Japanese adults reported significantly higher interdependence than older Americans, and that interdependence moderated the extent to which age-related interpretations were associated with functional health. Their findings are consistent with the view that relational orientation may be associated with the extent to which social representations of aging become consequential for the self (Levy et al., 2009). From this perspective, the coupling between other-directed VoA (in our study, the extent to which older adults are seen as resourceful vs. vulnerable) and self-directed VoA (in our study, the extent to which one views his or her own old age as resourceful vs. vulnerable) is theoretically meaningful because it indexes the degree to which socially shared representations are reflected in personal self-views, which is consistent with the internalization processes described by Rothermund and Brandtstädter (2003; see also the updated review by Park & Rothermund, in press).

Contemporary lifespan perspectives converge with stereotype-embodiment accounts in emphasizing that VoA are embedded in multilevel contexts and develop through transactions between persons and their social environments (Diehl & Wahl, 2024; Kornadt et al., 2022; Levy, 2009). Within such accounts, socially shared representations of aging are held to be more salient to self-definition where the self is habitually defined in relation to others (Markus & Kitayama, 1991). Interdependence therefore functions in the present study as a cultural-contextual anchor for the expectation that the strength of the association between other-directed and self-directed VoA may differ between the two countries; it is not advanced as a psychological process to be tested. The specific prediction that follows from this reasoning, and the reasons it is confined to the resources domain, are stated in the current study below.

Lifespan perspectives describe successful aging as the management of resources via selection, optimization, and compensation (Baltes, 1997; Moghimi et al., 2017), and conservation of resources theory emphasizes that perceived access to valued resources is pivotal for resilience, with resource loss and threatened loss carrying disproportionate psychological impact (Hobfoll et al., 2018). In addition, intergenerational tension is often framed in resource terms (e.g., succession and consumption domains of prescriptive ageism), making resources a culturally embedded axis of age-related evaluation (North & Fiske, 2013a, b). Resource-related content is therefore the axis along which later-life evaluation is most clearly articulated, and the one on which a difference in self–other coupling would be most interpretable.

### The Current Study

We conducted a cross-sectional, two-country comparative study in which older adults were recruited from middle- to high-socioeconomic-status assisted-living facilities in Israel and Japan and completed a questionnaire battery in Hebrew or Japanese following professional translation and back-translation. The study examined a four-facet VoA profile defined by target (self vs. other) and evaluative content (resources vs. vulnerability), together with independent and interdependent self-construal. Three hypotheses were tested. H1 predicted that Japanese participants would report higher interdependence than Israeli participants; independence was examined non-directionally. H2 predicted that country differences in VoA would be profile-specific rather than uniform, expressed in a Country × VoA-Facet pattern across Self-Resources, Self-Vulnerability, Other-Resources, and Other-Vulnerability. H3 predicted that the positive association between Other-Resources VoA and Self-Resources VoA would be stronger in Japan than in Israel.

Two features of H3 warrant explicit statement. First, Self-Resources VoA was specified as the primary outcome and Other-Resources VoA as the focal predictor, because resource-related content is the axis along which lifespan and intergenerational accounts of later life are most clearly articulated. The focused prediction therefore concerned whether the Other-Resources–Self-Resources association differed by country, rather than whether self–other coupling differed in every facet. Second, H3 is an interaction hypothesis about the structure of VoA rather than a mediation hypothesis. Interdependence is not hypothesized or tested as a psychological mediator; it serves as the cultural-contextual anchor described above for expecting the association to differ between the two countries. A stronger association in Japan would be consistent with accounts in which socially shared representations of aging are more salient to self-definition in more relational contexts (Markus & Kitayama, 1991). The study therefore does not establish stereotype internalization or identify interdependence as the process producing any country difference. Subjective health was included as a covariate in all models because it differed between the two national samples and was associated with the VoA indices.

A note on terminology is warranted, given that the design samples two countries rather than manipulating or measuring culture directly. Throughout, we use country to denote the sampled national contexts and the corresponding design variable, and we reserve cultural and cross-cultural for theoretical accounts and for the prior literature to which those accounts belong. Country in this design is a broad contextual proxy that subsumes cultural, institutional, historical, and socioeconomic differences, and the terminology is intended to keep that distinction visible.

## Method

### Participants

The dataset contained 390 records: 203 from Japan (52.1%) and 187 from Israel (47.9%). Age was available for 202 Japanese and 183 Israeli participants. Among those with age data, the mean age was 88.20 years in Japan (*SD* = 7.82, range = 63–107) and 85.21 years in Israel (*SD* = 5.55, range = 69–101), *t*(383) = 4.29, *p* <.001. Gender was available for all 203 Japanese participants and for 173 Israeli participants. Women comprised 149 of the 203 Japanese respondents (73.4%) and 134 of the 173 Israeli respondents with valid gender data (77.5%).

The two samples also differed on several sociodemographic characteristics. The proportion of participants with an academic education was significantly higher in Israel than in Japan (73.9% vs. 48.3%), χ²(1) = 24.73, *p* <.001. Significant differences were also found in marital status, χ²(3) = 23.30, *p* <.001: only 3.3% of participants in Israel had never married, compared with 17.9% in Japan, and the proportion of divorced participants was higher in Israel (6.7%) than in Japan (2.5%).

In addition, the mean number of children was significantly higher among Israeli than Japanese participants (*M* = 3.02, *SD* = 1.19 vs. *M* = 1.11, *SD* = 1.10), *t*(361) = 15.79, *p* <.001. Participants also rated their subjective health from 0 (poor health) to 100 (excellent health). Israeli participants reported higher subjective health than Japanese participants (*M* = 70.35, *SD* = 20.46 vs. *M* = 57.70, *SD* = 22.61), *t*(359) = 5.56, *p* <.001.

Given the significant differences in several background characteristics, as detailed in the Results section, the relevant characteristics were accounted for by including them as covariates in the statistical analyses. Table 1 presents the correlations between background variables and the main study variables within each sample.

**Table 1:** Means, standard deviations, and correlations among study variables in the Israeli (*n* = 187) and Japanese (*n* = 203) samples

Variable	M Israel	SD Israel	M Japan	SD Japan	1	2	3	4	5	6	7	8	9
1. Self-Resources VoA	3.52	0.75	2.50	0.75	—	0.02	0.68***	−0.04	−0.08	0.31***	0.18*	0.24***	0.17*
2. Self-Vulnerability VoA	2.09	0.98	2.26	0.83	−0.09	—	0.06	0.56***	0.04	−0.27***	−0.13	−0.12	0.01
3. Other-Resources VoA	3.04	0.69	2.55	0.67	0.50***	0.03	—	−0.02	0.01	0.32***	0.11	0.24***	0.17*
4. Other-Vulnerability VoA	2.90	0.88	2.56	0.73	0.16	0.51***	0.05	—	0.02	−0.24***	−0.09	−0.15*	0.06
5. Age	85.21	5.55	88.20	7.82	−0.02	0.06	−0.01	0.01	—	−0.05	−0.23**	−0.03	0.02
6. Subjective health	70.35	20.46	57.70	22.61	0.34***	−0.14	−0.01	0.06	−0.08	—	0.05	0.24***	0.16*
7. Education	5.17	0.99	4.46	0.77	0.11	−0.12	−0.05	0.09	−0.14	0.11	—	0.17*	0.09
8. Independence	4.95	1.25	4.78	1.04	0.12	−0.09	0.13	−0.08	−0.14	0.09	−0.13	—	0.68***
9. Interdependence	4.14	1.04	4.48	0.92	0.14	0.19	0.17*	0.18*	0.05	−0.01	−0.20*	0.23**	—

Israeli correlations are shown below the diagonal; Japanese correlations are shown above the diagonal. VoA = views on aging. Means and standard deviations are based on the available responses for each variable, and correlations were computed using pairwise available data; cell-specific ns therefore vary. Higher Resource scores indicate more positive views of aging, whereas higher Vulnerability scores indicate stronger vulnerability-related views of aging

* *p* <.05. ** *p* <.01. *** *p* <.001

### Measures

Sociodemographic Variables. To control for sociodemographic differences between the Israeli and the Japanese samples, we assessed chronological age, gender, marital status (single, married, divorced, widowed), education (graduate degree of MA and above, BA degree, completed high school, elementary–middle school, and no formal education), number of children and grandchildren, and perceived subjective health (on a visual analog scale ranging from 0 = poor health to 100 = excellent health).

Independence and Interdependence Scale. To control for cultural differences in relating to others and to the self, we used the 10-item Self-Construal Scale–Short Version (SCS-SV; D’Amico & Scrima, 2016) of the original Self-Construal Scale (SCS; Singelis, 1994). The abbreviated scale retains the original distinction between two theoretically separate dimensions. Five items assess independence, the tendency to view the self as autonomous and separate from others (e.g., “I do my own thing, regardless of what others think”), and five assess interdependence, the tendency to view the self as connected to and embedded within social relationships (e.g., “I will sacrifice my self-interest for the benefit of the group I am in”). Items were rated from 1 (strongly disagree) to 7 (strongly agree). The short form was suitable for the present cross-national study because it reduced respondent burden for advanced-age participants while retaining parallel assessment of the two self-construal dimensions across the Hebrew and Japanese versions. The SCS-SV was originally developed and validated in Italian university-student samples (D’Amico & Scrima, 2016) and, to our knowledge, had not previously been validated specifically among older Israeli or Japanese adults. Internal consistency was therefore examined separately in each national sample. An exploratory factor analysis (EFA) of the 10 items explained 45.95% of the total variance in the combined sample, 45.15% in the Israeli sample, and 53.74% in the Japanese sample. The Exploratory Factor Analysis (EFA) conducted on the current samples reconstructed independence (Cronbach’s α = 0.76 for the total sample, 0.79 for the Israeli sample, and 0.73 for the Japanese sample) and interdependence (Cronbach’s α = 0.61 for the total sample, 0.68 for the Israeli sample, and 0.59 for the Japanese sample), with five items loading on each factor.

Other- and Self-Views on Aging. Views on aging were assessed using two parallel five-item sets developed for the present cross-national study from longer descriptive aging-norm item pools (e.g., de Paula Couto et al., 2022; Wirth et al., 2023). Each set included five descriptive statements rated on a 5-point Likert scale ranging from 1 (strongly disagree) to 5 (strongly agree). The resources score comprised “healthy and fit,” “experienced and wise,” and “respected and honored,” and the vulnerability score comprised “lonely and isolated” and “emotionally vulnerable.” The distinction between self- and other-views was dictated by the instructions: “In my opinion, most older adults/I as an older adult am or will be …” The same descriptors were presented once with older adults in general as the target and once with the respondent’s own aging self as the target. The binational research team selected the descriptors because they aligned with current theoretical approaches to views on aging. Those approaches distinguish self-directed views from views of older adults in general (Rothermund & de Paula Couto, 2024; Shrira et al., 2022) and recognise the coexistence of resource- or gain-related and vulnerability- or loss-related content in aging-related views (Charles, 2010; Kornadt et al., 2020). The descriptors also permitted parallel self/other wording. The resulting brief, parallel format reduced respondent burden and facilitated close semantic matching across the Hebrew and Japanese versions. Because these parallel five-item sets were developed for the present study, they had not previously undergone psychometric validation in older Israeli or Japanese populations. Principal-axis factoring with direct-oblimin rotation and a specified two-factor solution, using listwise deletion, was conducted in the pooled sample and separately by country. The EFA identified Other-Resources (3 items) and Other-Vulnerability (2 items), accounting for 44.13% of the variance. Parallel self-view factors accounted for 54.38% of the variance overall (41.82% in Israel; 56.80% in Japan). Internal consistency was marginal to high: for Other-Resources VoA, Cronbach’s α = 0.61 for the total sample, 0.49 for the Israeli sample, and 0.73 for the Japanese sample; for Other-Vulnerability VoA, α = 0.64, 0.61, and 0.69, respectively; for Self-Resources VoA, α = 0.75, 0.54, and 0.76, respectively; and for Self-Vulnerability VoA, α = 0.68, 0.69, and 0.72, respectively. Scores were means of the relevant items, with higher resource scores indicating stronger resource-related VoA and higher vulnerability scores indicating stronger vulnerability-related VoA. These EFAs and reliability estimates provide preliminary within-sample structural and internal-consistency information but do not establish criterion validity, test–retest reliability, or cross-national measurement invariance.

### Procedure

Participants in Israel and Japan were recruited from middle- to high-socioeconomic-status assisted-living facilities. In Israel, recruitment occurred in two major cities in central Israel; in Japan, recruitment occurred in the Kyoto area. Facility residents volunteered to participate, questionnaire completion took approximately 40 min, and eligibility required staff confirmation that participants had no diagnosis of dementia and had sufficient Hebrew or Japanese proficiency to complete the questionnaires. In each country, locally based research assistants received standardized instructions on eligibility screening, consent, neutral questionnaire administration, and responding to participant questions without influencing responses. No financial or material incentive was provided. Participants were told that participation was voluntary and that they could stop the questionnaire at any time. No participant subsequently requested removal of their data. Because this was a one-session cross-sectional survey, there was no longitudinal follow-up or longitudinal attrition. Some records contained missing responses. Consistent with the analysis syntax and dataset, missing data were handled by listwise deletion within each analysis, resulting in analysis-specific sample sizes.

## Results

Data were analyzed using IBM SPSS Statistics 28. Initial associations were examined using Pearson correlations, and independent correlations were compared using Fisher’s r-to-z transformation. Country differences in self-construal and in the four VoA facets were tested with mixed analyses of covariance. H3 was tested with hierarchical regression, and the focused conditional-effects model was estimated with PROCESS Version 4.2 (Model 1; Hayes, 2022). For the hierarchical regression, tolerance values ranged from 0.28 to 0.79 and variance inflation factors (VIFs) ranged from 1.26 to 3.57, indicating no severe multicollinearity (O’Brien, 2007).

### Preliminary Analyses and Descriptive Statistics

The dataset contained 390 records (Israel: *n* = 187; Japan: *n* = 203). Missing data were handled using listwise deletion within each analysis. Therefore, sample sizes varied across measures. For the primary study variables, valid *N*s ranged from 351 (Interdependence) to 385 (Age), and the relevant analysis-specific *N* is reported with each result. No formal a priori power analysis was conducted. Recruitment was based on the eligible residents available in the participating facilities. A sensitivity calculation based on the realized country samples (Israel *n* = 187; Japan *n =* 203; two-sided α = 0.05) indicated 80% power to detect a between-country standardized mean difference of approximately *d* = 0.285. For the focused one-interaction regression (*N* = 344), the corresponding 80%-power threshold was an incremental f² of approximately 0.023. The observed Country × Other-Resources interaction accounted for ΔR² = 0.005.

To assess distributional properties and identify potential violations of normality assumptions, skewness and kurtosis indices were examined for the primary study variables (Self-Resources, Self-Vulnerability, Other-Resources, Other-Vulnerability, Independence, and Interdependence). Given the sample size, the evaluation relied on these indices rather than on formal tests of normality. Across the full sample, all skewness and kurtosis values for the primary variables were below 1 in absolute value, indicating no substantial deviations from normality (West et al., 1995).

Pearson correlations among the aging-perception indices and background variables were computed separately for the Israeli and Japanese samples (Table 1). Self-Resources VoA was positively associated with Other-Resources in both countries (Israel: *r* =.50, *n* = 182; Japan: *r* =.68, *n* = 190). A Fisher r-to-z comparison showed that these independent correlations differed significantly, *z* = 2.66, *p* =.008, with a stronger association in Japan. Self-Vulnerability VoA was also positively associated with Other-Vulnerability in both samples (Israel: *r* =.51, *n* = 176; Japan: *r* =.56, *n* = 190), but this country difference was not significant, *z* = 0.74, *p* =.459. Cross-domain associations were small and nonsignificant (Self-Resources with Other-Vulnerability: Israel *r* =.16, Japan *r* = −.04; Self-Vulnerability with Other-Resources: Israel *r* =.03, Japan *r* =.06). The resource-domain country difference was further evaluated in the adjusted moderation analyses reported below.

Associations of aging perceptions with subjective health were generally in the expected directions but differed in magnitude across countries. In Israel, subjective health was positively correlated with Self-Resources (*r* =.11). It showed weak and nonsignificant negative associations with Self-Vulnerability (*r* = −.14) and Other-Vulnerability (*r* = −.05), whereas its association with Other-Resources was small (*r* =.09). In Japan, subjective health showed stronger and more systematic associations, including positive correlations with Self-Resources (*r* =.32) and Other-Resources (*r* =.31), and negative correlations with Self-Vulnerability (*r* = −.24) and Other-Vulnerability (*r* = −.27; see Table 1).

### Analyses of Independence and Interdependence in the Two Study Groups

To test whether Independence and Interdependence differed by country, a mixed analysis of covariance was conducted with country (Israel vs. Japan) as the between-subjects factor and self-construal dimension (Independence vs. Interdependence) as the repeated measure. Age and education were initially examined but were not retained because they were nonsignificant. The final model controlled for standardized subjective health. The analysis included 162 Israeli and 175 Japanese participants. Descriptive means were Independence *M* = 4.95 (*SD* = 1.27) and Interdependence *M* = 4.13 (*SD* = 1.06) in Israel, and Independence *M* = 4.78 (*SD* = 1.04) and Interdependence *M* = 4.48 (*SD* = 0.92) in Japan. Subjective health was significant, *F*(1, 334) = 7.86, *p* =.005, ηp² = 0.02. The country main effect was not significant, *F*(1, 334) = 2.82, *p* =.094, ηp² = 0.01. The self-construal-dimension effect, *F*(1, 334) = 78.66, *p* <.001, ηp² = 0.19, and the Country × Self-Construal Dimension interaction, *F*(1, 334) = 11.71, *p* =.001, ηp² = 0.03, were significant.

Follow-up tests showed that Independence exceeded Interdependence in both Israel and Japan (both *ps* < 0.001), with a larger difference in Israel. Independence did not differ significantly between countries, *F*(1, 334) = 0.16, *p* =.688, ηp² < 0.001, whereas Interdependence was higher in Japan than in Israel, *F*(1, 334) = 12.42, *p* <.001, ηp² = 0.04. Thus, H1 was supported for Interdependence, while the non-directional comparison of Independence showed no significant country difference.

### Differences Between Perceptions of Others and Self-Perceptions of Aging

**Fig. 1:**
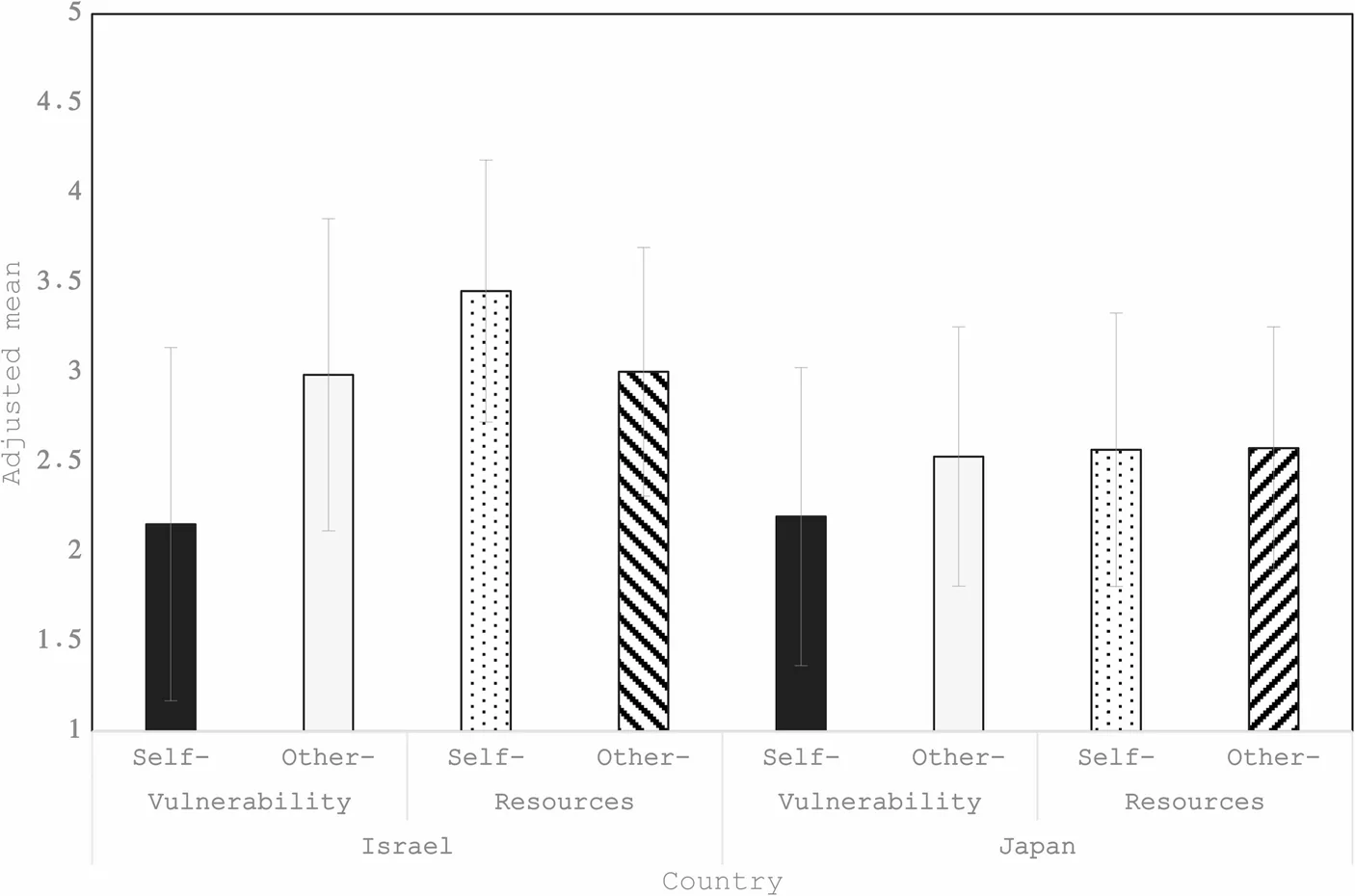
Estimated marginal means of the four views-on-aging (VoA) facets by country. Values are estimated marginal means adjusted for standardized subjective health (Israel *n* = 164; Japan *n* = 185); error bars represent ± 1 SD of the observed complete-case scores. These adjusted means are not identical to the observed means reported in the text and in Table 1. Bar fills distinguish the four facets, which are also identified on the category axis. Higher Resource scores indicate more positive views of aging, whereas higher Vulnerability scores indicate stronger vulnerability-related views of aging

To examine whether country differences varied across the four VoA facets, a mixed analysis of covariance was conducted with country (Israel vs. Japan) as the between-subjects factor and VoA facet (Self-Resources, Self-Vulnerability, Other-Resources, and Other-Vulnerability) as the repeated measure. Standardized subjective health was included as a covariate. The analysis included 164 Israeli and 185 Japanese participants. The means and standard deviations reported below are observed descriptive values among these complete cases and therefore differ slightly from the full-sample descriptive statistics in Table 1, which are based on pairwise available data. The inferential tests adjusted for subjective health, and Fig. 1 displays the corresponding estimated marginal means. In Israel, means were 3.54 (*SD* = 0.73) for Self-Resources, 2.09 (*SD* = 0.98) for Self-Vulnerability, 3.05 (*SD* = 0.69) for Other-Resources, and 2.95 (*SD* = 0.87) for Other-Vulnerability. In Japan, corresponding means were 2.50 (*SD* = 0.76), 2.25 (*SD* = 0.83), 2.54 (*SD* = 0.68), and 2.56 (*SD* = 0.72). The Subjective Health × VoA Facet effect was significant, *F*(3, 344) = 17.48, *p* <.001, ηp² = 0.13. The country main effect, *F*(1, 346) = 59.18, *p* <.001, ηp² = 0.15, and the Country × VoA Facet interaction, *F*(3, 344) = 24.55, *p* <.001, ηp² = 0.18, were also significant (see Fig. 1).

Follow-up repeated-measures analyses were conducted separately within each country. The four VoA facets differed in Israel, *F*(3, 160) = 59.46, *p* <.001, ηp² = 0.53, and in Japan, *F*(3, 181) = 14.85, *p* <.001, ηp² = 0.20. In Israel, Self-Resources exceeded each of the other three facets (all *ps* < 0.001), Self-Vulnerability was lower than Other-Resources and Other-Vulnerability (both *ps* < 0.001), and Other-Resources did not differ from Other-Vulnerability (*p* =.333). In Japan, Self-Vulnerability was lower than Self-Resources, Other-Resources, and Other-Vulnerability (all *ps* < 0.001). The remaining three facets did not differ significantly from one another (*ps* = 0.244–0.563). Country contrasts, adjusted for subjective health, showed higher Self-Resources, Other-Resources, and Other-Vulnerability in Israel than in Japan (all *ps* < 0.001), whereas Self-Vulnerability did not differ (*p* =.664).

To evaluate robustness, a supplementary model added standardized Independence and Interdependence as covariates alongside subjective health (Israel *n* = 162; Japan *n* = 182). The Country × VoA Facet interaction remained significant, *F*(3, 337) = 21.13, *p* <.001, ηp² = 0.16. Adjusted country contrasts retained the same pattern: Israel was higher on Self-Resources, Other-Resources, and Other-Vulnerability (all *ps* < 0.001), whereas Self-Vulnerability did not differ (*p* =.600). Thus, the profile interaction and its directional interpretation were not reversed by adjustment for the self-construal scores.

Taken together, the primary and supplementary models supported a profile-specific rather than uniform country difference. The most consistent pattern was higher Self-Resources and Other-Resources in Israel, accompanied by higher Other-Vulnerability, while Self-Vulnerability did not differ significantly between countries. Because higher vulnerability scores indicate more vulnerability-related VoA, the resource and vulnerability contrasts should not be collapsed into a single positive-versus-negative country ranking.

### Country Differences in the Relations Between Self- and Other-Related Views on Aging

The third hypothesis was examined by hierarchical regression with standardized Self-Resources VoA as the dependent variable. In Step 1, standardized subjective health and country (Israel = 1, Japan = 0) were entered. In Step 2, the standardized main effects of Other-Resources, Other-Vulnerability, and Self-Vulnerability were added. In Step 3, the Country × Self-Vulnerability, Country × Other-Resources, and Country × Other-Vulnerability products were entered. The complete-case sample was *N* = 349 (Israel *n* = 164; Japan *n* = 185; see Table 2).

**Table 2:** Hierarchical regression predicting self-resources views on aging (*N* = 349)

Predictor	B	SE	β	*R*²	ΔR²
*Step 1*				0.394	0.394
(Intercept)	−0.484***	0.059	—		
Subjective health	0.281***	0.045	0.274		
Country	0.977***	0.088	0.487		
*Step 2*				0.599	0.205
Self-Vulnerability VoA	−0.076	0.042	−0.075		
Other-Resources VoA	0.483***	0.037	0.486		
Other-Vulnerability VoA	0.100*	0.042	0.099		
*Step 3*				0.612	0.013
Country × Self-Vulnerability VoA	−0.203*	0.082	−0.150		
Country × Other-Resources VoA	−0.139	0.073	−0.094		
Country × Other-Vulnerability VoA	0.199*	0.085	0.146		

*N* = 349 (Israel *n* = 164; Japan *n* = 185). VoA = views on aging; *B* = unstandardized regression coefficient; *SE* = standard error; β = standardized regression coefficient. The dependent variable and all continuous predictors were standardized before analysis; country was coded 0 = Japan and 1 = Israel. Step 1: *F*(2, 346) = 112.34, *p* <.001, *R²* = 0.394. Step 2: *ΔF*(3, 343) = 58.60, *p* <.001, *ΔR²* = 0.205, total *R²* = 0.599. Step 3: *ΔF*(3, 340) = 3.87, *p* =.010, *ΔR²* = 0.013, final *R²* = 0.612 (adjusted *R²* = 0.603), *F*(8, 340) = 67.15, *p* <.001. The Country × Other-Resources interaction is estimated here alongside two other product terms; the primary test of H3 is the focused conditional-effects model reported in the text

* *p* <.05. ** *p* <.01. *** *p* <.001

As shown in Table 2, subjective health and country jointly accounted for R² = 0.394 in Step 1, *F*(2, 346) = 112.34, *p* <.001. Better subjective health was associated with higher Self-Resources VoA, and Self-Resources VoA was higher in Israel than in Japan.

In Step 2, Other-Resources VoA was positively associated with Self-Resources VoA (*p* <.001), Other-Vulnerability was also positively associated with it (*p* <.05), and Self-Vulnerability was not significant. This block increased explained variance by ΔR² = 0.205, Δ*F*(3, 343) = 58.60, *p* <.001, yielding R² = 0.599 (adjusted R² = 0.593).

In Step 3, the Country × Self-Vulnerability interaction was significant, *B* = − 0.203, *SE* = 0.082, β = −0.150, *t*(340) = − 2.48, *p* =.014, 95% CI [− 0.364, − 0.042]. The Country × Other-Vulnerability interaction was also significant, *B* = 0.199, *SE* = 0.085, β = 0.146, *t*(340) = 2.35, *p* =.020, 95% CI [0.032, 0.366]. The Country × Other-Resources interaction did not reach significance in this specification, *B* = − 0.139, *SE* = 0.073, β = −0.094, *t*(340) = − 1.90, *p* =.058, 95% CI [− 0.282, 0.005]. The interaction block added ΔR² = 0.013, Δ*F*(3, 340) = 3.87, *p* =.010. The final model explained R² = 0.612 (adjusted R² = 0.603), *F*(8, 340) = 67.15, *p* <.001.

To examine the robustness and interpretability of the resource-domain interaction, a focused conditional-effects analysis was conducted using PROCESS Version 4.2 (Model 1; *N* = 344). Country was specified as the independent variable and Other-Resources VoA as the moderator, controlling for subjective health, Independence, Interdependence, Self-Vulnerability, and Other-Vulnerability. This focused model followed the resource-specific H3 and retained the constituent main effects required to interpret the interaction.

**Fig. 2:**
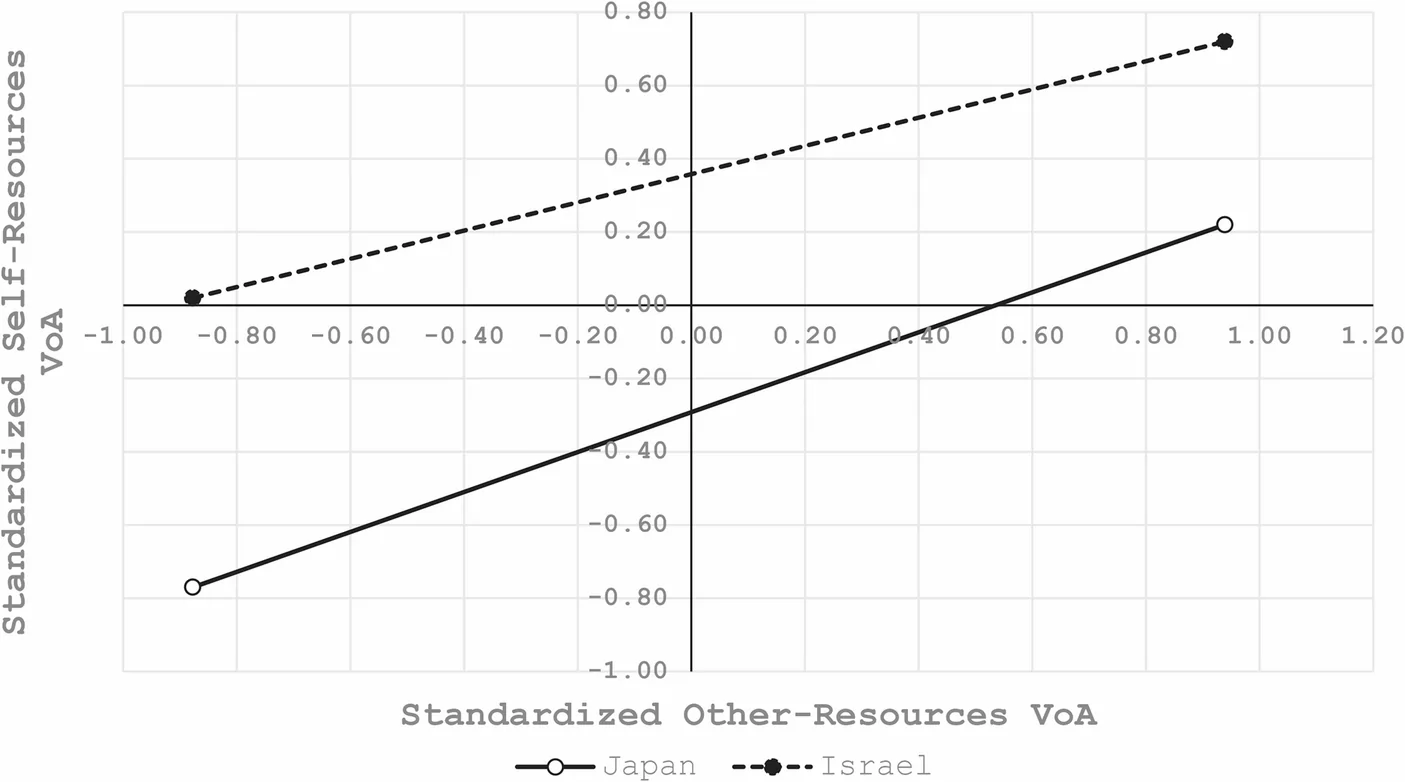
Country moderation of the association between Other-Resources VoA and Self-Resources VoA. Lines show model-predicted standardized Self-Resources VoA across the plotted range of standardized Other-Resources VoA (*N* = 344), controlling for subjective health, Independence, Interdependence, Self-Vulnerability, and Other-Vulnerability. Solid line = Japan; dashed line = Israel

The Country × Other-Resources VoA interaction was significant, *B* = − 0.15, *SE* = 0.08, *t*(335) = − 2.05, *p* =.042, 95% CI [− 0.30, − 0.01], accounting for *ΔR²* = 0.005 beyond the main effects and covariates. Conditional effects are shown in Fig. 2. Self-Resources VoA increased with Other-Resources in both countries, but the association was steeper in Japan than in Israel.

The two analyses differ in three respects. The hierarchical model estimated all three Country × VoA product terms simultaneously in a complete-case sample of 349, whereas the focused model estimated the single pre-specified Country × Other-Resources interaction in a complete-case sample of 344 and additionally adjusted for Independence and Interdependence. Because the three product terms were themselves correlated, entering them together distributed the shared resource-domain variance across them and reduced the precision of each. The coefficients were nevertheless close in magnitude and identical in direction (*B* = − 0.14 and *B* = − 0.15), and the two p values fall on either side of 0.05 rather than pointing to different conclusions. Because H3 was specified in advance as a resource-domain hypothesis with a single interaction, the focused conditional-effects model is treated as the primary test, and the three-interaction block in Table 2 is reported as a secondary, more conservative specification.

The focused adjusted model therefore supported H3 in the resources domain, although the incremental effect was small. The additional vulnerability-related interaction coefficients in the broader hierarchical model were not stable across specifications and were not treated as primary evidence for H3. The most defensible conclusion is that the Other-Resources–Self-Resources association was stronger in Japan than in Israel in both the raw-correlation comparison and the focused adjusted moderation analysis.

## Discussion

VoA can refer to one’s own aging as well as to older adults in general and can contain both resource- and vulnerability-related content (Park & Rothermund, in press; Rothermund & de Paula Couto, 2024). The present study examined these four facets in assisted-living samples from Japan and Israel. Japanese participants reported higher interdependence than Israeli participants, whereas independence was examined without a directional prediction and did not differ significantly. Country differences in the VoA facets remained evident after adjustment for subjective health and, in the supplementary model, self-construal variables. These findings indicate country-related patterning that is not statistically reducible to the measured self-construal scores. They do not establish self-construal as the mechanism producing the country differences.

Country differences in VoA were facet-specific rather than uniformly positive or negative, consistent with cross-cultural work showing that aging attitudes vary across domains and operationalizations (Fung, 2024; Löckenhoff et al., 2009). Self-Vulnerability was the lowest facet in both country-specific profiles. Israeli participants reported higher Self-Resources, Other-Resources, and Other-Vulnerability than Japanese participants, whereas Self-Vulnerability did not differ significantly between countries. The same pattern of country differences remained after adding Independence and Interdependence as covariates. Thus, the findings cannot be summarized as one country holding more positive or more negative VoA globally.

The four-facet framework reveals a pattern that a global VoA score would obscure: Israel was higher on both resource facets but also on Other-Vulnerability, while Self-Vulnerability was similar across countries. This combination demonstrates why resources and vulnerability should not be treated as opposite endpoints of a single country-level positivity continuum. Nevertheless, the brief indices showed variable internal consistency, particularly in the Israeli resource scales, so the exact profile should be considered preliminary.

The formal moderation analysis provided qualified support for H3 in the resources domain. Other-Resources VoA was positively associated with Self-Resources in both countries, and the Country × Other-Resources interaction indicated a steeper association in Japan after adjustment for subjective health, Independence, Interdependence, and the other VoA main effects. This result is consistent with the possibility that socially directed resource representations are more closely aligned with aging self-views in the Japanese sample. However, the study did not measure stereotype internalization and cannot determine whether relational self-construal, social norms, response processes, or another country-linked factor produced the interaction.

The magnitude of these effects should temper their interpretation. In the hierarchical regression predicting Self-Resources VoA, the interaction block added a small though statistically significant increment in explained variance, and the focused conditional-effects model accounted for only a small additional proportion of variance beyond the main effects and covariates (both reported in the Results). These small increments, together with the measurement limitations and the dependence of the estimates on the covariates included, warrant caution despite the converging raw-correlation and adjusted-interaction results, and the resource-domain moderation should be treated as requiring replication.

The present data do not explain why the Other-Resources–Self-Resources association differed across countries. Norms concerning dependence, burden, aging in place, or appropriate resource use may be relevant contextual hypotheses, but none was measured here. They therefore cannot be treated as explanations of the observed interaction. Future studies should assess these proposed norms directly and test whether they account for country differences in self–other coupling.

Accordingly, applied implications should remain tentative. The current findings do not establish that resource-based messaging or interventions would have different effects in Japan and Israel. They instead identify a resource-focused self–other association that can be examined prospectively and experimentally in future cross-cultural research.

A key strength of this study is its two-country design, focusing on older adults in middle- to high-socioeconomic-status assisted-living facilities in Japan and Israel, which enables a targeted comparison of VoA structure across two national contexts. Conceptually, we operationalized VoA within a four-facet framework (self/other × resources/vulnerability), enabling profile-based interpretation rather than reducing VoA to a single positivity score. In this way, we used an approach that is consistent with contemporary views of VoA as multidimensional (Klusmann et al., 2020). Another strength is the use of the residents’ native language after professional translation and back-translation.

Several limitations should be noted. First, the cross-sectional design does not establish temporal direction, stereotype internalization, mediation by interdependence, or any of the contextual mechanisms discussed above. Country is a broad contextual indicator and cannot isolate cultural norms from institutional, historical, socioeconomic, or response-process differences. Second, both samples were drawn from middle- to high-socioeconomic-status assisted-living facilities, restricting socioeconomic and functional variability and limiting generalizability to community-dwelling older adults and residents of other care settings. Third, neither the SCS-SV nor the parallel five-item VoA sets had previously been validated specifically among older Israeli or Japanese adults. Although the analyses conducted in the present samples provide preliminary evidence concerning factor structure and internal consistency, they do not constitute full psychometric validation. Fourth, internal consistency varied across the brief measures. Particularly low coefficients were found for Other-Resources and Self-Resources VoA in Israel, for Interdependence in the total sample and in Japan, and for several other coefficients. Because the VoA subscales contained only two or three items, their alpha coefficients were partially affected by the small number of items (Cortina, 1993; Schmitt, 1996). Low reliability attenuates observed associations and adds error to group means, so it may have affected both the mean-level country comparisons and the estimated associations reported here; this concern applies most directly to the Israeli Other-Resources and Self-Resources scales and to the Interdependence scale, which showed the lowest coefficients. Measurement error is important in cross-cultural comparisons, where differences across language and cultural groups can complicate the interpretation of observed mean differences (Chen, 2008; Fischer & Karl, 2019; Putnick & Bornstein, 2016). Therefore, findings involving the lower-reliability indices should be regarded as preliminary and should be replicated using longer, culturally validated measures. Fifth, separate exploratory factor analyses recovered similar intended factors in each country, but this does not establish configural, metric, or scalar invariance. Scalar equivalence, which is especially important for mean comparisons, was not established. Reference-group standards and response-style differences may also affect cross-cultural self-report comparisons (Heine et al., 2002; Milfont & Fischer, 2010). Thus, mean-level findings should be interpreted as provisional country-specific profiles, and the moderation finding requires replication with longer, culturally validated measures and explicit measurement-invariance testing. Finally, no formal a priori power analysis was conducted.

Future work should (a) use longitudinal cross-lagged designs to test directionality between resource- and vulnerability-based VoA, (b) recruit more diverse, community-based samples to evaluate generalizability beyond high-end residential settings, and (c) conduct more explicit measurement-invariance and response-style checks to strengthen cross-national inference (Milfont & Fischer, 2010). Linking VoA facets to behavioral and health outcomes (e.g., functional capacity, healthcare use, activity engagement) would clarify the practical significance of self–other coupling in the resources domain (Kornadt et al., 2020). Finally, extending comparisons beyond Japan and Israel can test whether the observed moderation reflects interdependence specifically or broader socio-structural forces shaping how socially shared age narratives map onto personal self-views (Diehl & Wahl, 2024; Kornadt et al., 2020).

In sum, the findings support treating VoA as a target- and content-specific profile rather than as a single evaluative score. The most consistent moderation result indicated that Other-Resources VoA was more strongly associated with Self-Resources in Japan than in Israel under the adjusted model. This pattern is compatible with cultural-contextual accounts of self–other alignment but does not establish the mechanism responsible for it. Replication with longer invariant measures, broader residential and community samples, longitudinal designs, and direct measures of the proposed cultural mechanisms is needed.

## Data Availability

De-identified data and analysis code will be shared upon reasonable request to the corresponding author.

## Declarations

### Use of AI-assisted technologies in the writing process

During the preparation of this revision, the authors used Claude (Anthropic) to assist in organizing the final response to the editor and reviewers. Grammarly was used for additional language editing. No AI tool was used to generate or analyze data, and no AI tool contributed original scientific claims, interpretations, or conclusions. The authors reviewed, verified, and edited all output and take full responsibility for the publication's content.
